# “He Who Dines with the Leopard Is Liable To Be Eaten”

**DOI:** 10.3201/eid1209.AC1209

**Published:** 2006-09

**Authors:** Polyxeni Potter

**Affiliations:** * Centers for Disease Control and Prevention, Atlanta, Georgia, USA

**Keywords:** tingatinga, leopard, tanzania, african art, bicycle paint, Masonite

Proverb of the Fipa, southwest Tanzania

Bicycle paint—thick, slow to dry, and available—proved just the right medium for working on masonite, canvas of choice for tingatinga art. The concept originated with Edward Saidi Tingatinga (1932–1972), a Mozambican who lived and worked in Tanzania. Tingatinga had scant opportunity for artistic training or academic pursuits. Growing up in a farm family, he was preoccupied with survival, doing odd jobs to escape poverty. Talented nonetheless and energetic and living in East Africa, a region rich in artistic traditions (music, poetry, dance, sculpture), he drifted toward creative work: music at first, then embroidery, weaving, and house paintings—a type of continuous decoration wrapped around dwellings, forcing the viewer to circle the work to experience it fully (*1*). In the 1960s, he started painting animals and other single motifs in a consistent and colorful but spare style.

Tingatinga's preference for bicycle paint involved more than availability. Because it dried slowly, the paint required separate processing of the background before other elements could be superimposed, creating sharp color contrasts and adding to the abstract quality of the work. The characteristic consistency also facilitated shading and color variation (*2*).

The paintings were taken to Oyster Bay, an affluent section of Dar es Salaam, Tanzania's commercial capital, and sold well at the Morogoro stores, which catered to visitors. The boldness and excitement of the work attracted attention. Tingatinga was invited to join the National Arts Company and was able to devote all his time to painting. He soon employed several apprentices, who eventually produced independent work in the same style (*3*). He died young, but his work, continued by his relatives and students, burgeoned into a local genre, widely imitated and identified with the region. The art became known beyond Tanzania, in Kenya and other parts of Africa, as well as Norway, Sweden, Finland, Denmark, and other parts of Europe. It was put on display in Tanzania's national museum, the first domestic exhibition to be included and the first by a self-taught artist.

Tingatinga painted a theme prominent in southern and eastern African art, "the big five" large animals (elephant, lion, rhinoceros, buffalo, leopard) common in this region. "Naïve" but intuitively in touch with the elements of this and other African themes, the artist chose vibrant color and abstraction over naturalistic description, representing rather than depicting his subjects. This element of abstraction in African art (often seen in traditional masks or wood, ivory, stone, and other carvings), as well as reliance on bold dramatic color, was extremely influential in the development of modern art, inspiring such masters as Pablo Picasso, Henri Matisse, and Amadeo Modigliani (*4*).

Tingatinga's work contained elements of the Makonde and Swahili cultures and coastal East African design. A celebration of nature and ordinary daily activities, it was characterized by borderless motifs and direct presentation. Seemingly uncomplicated, it was symbolic and metaphorical, using content, color, and design to represent beliefs and ideas. Now as ubiquitous as visitors to Africa, tingatinga art is widely reproduced on anything from small ceramic tiles to large masonite squares and is part of the global "tourist" or "airport trade," prompting some to question its authenticity.

The leopard, on this issue's cover, was painted by Daudi, Tingatinga's son, who has been recreating some of his father's most representative works (*5*). The ferocious, carnivorous mammal it represents is stealthy and shrewd, solitary, and loyal only to its cubs. Eschewing a tawny coat, this one wears black and sports the circular rosettes of East African leopards. Awash in the primary colors of the region's Bantu societies: red, white, and black—triad of the spirit world (e.g., black for African; white for Arab, European; red for spirits of the dead) (*6*), it dominates the painting from edge to edge, soft on its toes, ready to leap.

The zoologic profile—appearance as well as behavior of this wild cat—illuminates its symbolism, so prevalent in African cosmology and lore. Much has been made of the leopard's maternal bias and high, yet secondary (to the lion), position in the animal kingdom. Its speckled black-and-white pelt has stood for contrast: day and night, wet and dry, human and wild. Its unpredictable temperament and trickery are symbols of, among other topical scourges, despotic rule. Symbolism extends to leopard skin garments and the power of those who wear them, whether heroic figures, political leaders, or spiritual healers (*7*).

Symbolic elaboration of the leopard, old as enigmatic spots on prehistoric caves and diverse as variegated fur, does not fail to apply to emerging diseases. And the subtle nuances of Tingatinga's intuitive expression capture more than Africa's artistic elements. The expansion of human communities into the wild, a factor in disease emergence, is rampant on all continents, including Africa. The proximity of human and animal habitation, the constant interaction—amicable, hostile, parasitic—and human fascination with the wild place us at the proverbial table with the leopard to partake at our own risk.

**Figure:**
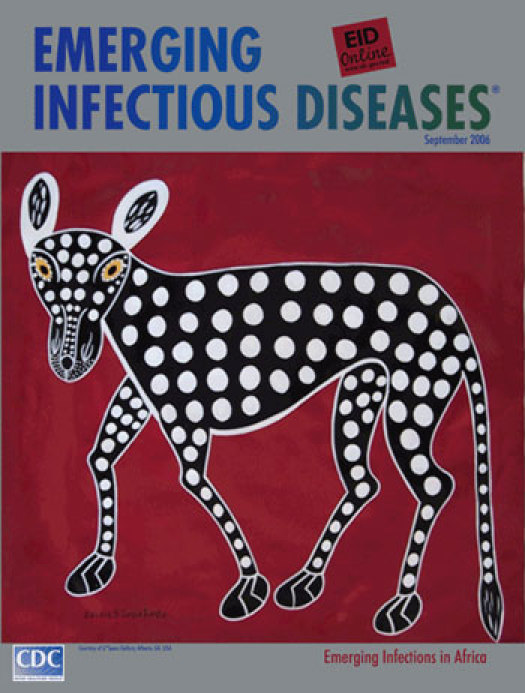
Daudi E.S. Tingatinga. Leopard (2006). Acrylic bicycle paint on canvas (73.66 cm × 73.66 cm) Courtesy of U*Space Gallery (http://www.uspacegallery.com), Atlanta, Georgia, USA
